# Post-migration stressors, mental health and well-being in resettled refugees from Syria: Do individuals’ coping strategies matter?

**DOI:** 10.1186/s13031-023-00556-3

**Published:** 2023-12-20

**Authors:** Øivind Solberg, Alexander Nissen, Fredrik Saboonchi

**Affiliations:** 1 Department of Health Sciences, The Swedish Red Cross University College, Huddinge, Sweden; 2 Division for Forced Migration and Refugee Health, Norwegian Centre for Violence and Traumatic Stress Studies, Oslo, Norway; 3 Division of Insurance Medicine, Department of Clinical Neuroscience, Karolinska Institutet, 171 77 Stockholm, Sweden

**Keywords:** Refugees, Mental health, Anxiety, Depression, Well-being, Coping, Post-migration stress

## Abstract

**Background:**

The evidence is mixed as to whether individuals’ coping strategies may mitigate the adverse mental health effects of post-displacement stressors in refugee populations, with some indications that the buffering effects of coping strategies are context dependent. The present study examined if problem-solving and acceptance coping strategies were effect modifiers between post-migration stressors and mental health in adult refugees from Syria resettled in Sweden.

**Methods:**

Study aims were investigated using cross-sectional survey data from a nationwide, randomly sampled group of adult refugees from Syria granted permanent residency in Sweden between 2011 and 2013 (N_sample_ = 4000, n_respondents_ = 1215, response rate 30.4%). Post-migration stressors examined included: financial strain, social strain, host-country competency strain and discrimination. Two mental health outcomes were used: anxiety/depression, measured with the Hopkins Symptom Checklist-25; and well-being, measured with the WHO-5 Well-being Index. Both outcomes were modelled continuously. Coping strategies were measured using the BRIEF Cope scale. Interactions between coping strategies and post-migration stressors were tested in fully adjusted linear regression models using Wald test for interaction, corrected for multiple testing using the Benjamini–Hochberg procedure.

**Results:**

Both problem-solving and acceptance coping strategies buffered the adverse association between financial strain and symptoms of anxiety/depression, and problem-solving coping strategies buffered the adverse association between host-country competency strain and anxiety/depression.

**Conclusions:**

The study suggests that individuals’ coping strategies may to some degree buffer the adverse mental health effects of financial strain and host-country competency strain experienced by refugees in the resettlement phase. Although this pattern was only found in regard to anxiety/depression and not subjective well-being, the findings show that individual-level coping skills among refugees may contribute to adaptation in the face of post-settlement adversities. Notwithstanding the importance of attending to refugees’ psychosocial conditions, refugees residing in refugee camps and newly resettled refugees might benefit from interventions aiming at enhancing individual coping resources and skills. The potential effect of increased controllability and decreased conflict-proximity also warrants further exploration in future studies.

## Introduction

According to the United Nations’ High Commissioner for Refugees (UNHCR), 110 million people are currently forcibly displaced [1]. Of these 110 million, it is estimated that 36.4 million are refugees. Forced migration of refugees to neighbouring countries, including Europe and Scandinavia, accentuate the need for research that focuses on how to best receive and care for refugees in the resettlement phase and help refugees cope with the negative psychological consequences of forced migration.

Previous research has indicated that refugees from e.g., Syria report heightened symptoms of depression and posttraumatic stress [e.g., 2–4] and reduced health-related quality of life as a consequence of exposure to potentially traumatic pre-, peri- or post-flight experiences [5]. Research has also demonstrated that several common coping strategies, e.g., cognitive coping, religious coping, and/or emotional coping, are utilized in attempts to alleviate distress related to forced migration [6–9]. Coping strategies are typically known as individual approaches, skills and/or abilities commonly utilized to manage psychosocial stressors in order to prevent and/or minimize stress or illness [10, 11]. Conversely, using unhelpful coping strategies (e.g., avoidance and rumination) has also been shown to maintain prolonged grief and heightened symptoms of post-traumatic stress disorder (PTSD) and/or depression among bereaved adults [12].

However, previous research has also shown that the use of coping strategies that are known in the literature to be generally adaptive, e.g., problem-focused coping strategies, can sometimes have the opposite effect, indirectly increasing symptoms of depression and anxiety, as found in a study of Syrian refugees living in refugee camps near the Syrian border [13]. In this study, authors found that increased symptoms of anxiety and depression was mediated by problem-focused coping strategies when the relationship between self-regulatory systems (promotion focus/prevention focus) and mental health symptoms was examined, demonstrating that problem-focused coping might be maladaptive in certain contexts.

On the other hand, Woltin and colleagues did not find the same results in a similar Syrian refugee sample in a refugee camp in Germany [13]. In this more distal context, problem focused coping had a positive effect, alleviating symptoms of depression and anxiety. Woltin and colleagues explain this by alluding to the fact that coping might be effectful in the German, refugee camp-context, as conflict zone proximity (along with other contextual factors) is more distal, leading to less distress and that the situation is being perceived as more controllable or solvable, thus not undermining the adaptiveness of problem-focused coping, as might be the case in the more proximal context by the Syrian border. In the authors’ view, this suggests that when stressors are being subjectively perceived as uncontrollable or unchangeable, problem-focused coping can be detrimental, and that a more disengaged, emotion-focused coping could be more adaptive in such a context [13].

In a similar vein, a previous study conducted by our research group on adult asylum-seekers from different countries (e.g., Afghanistan, Syria, Somalia, Iraq and Eritrea) in Sweden found no evidence of a buffering effect of coping in the association between post-migratory stressors and wellbeing [14]. In this study, we argue that the “limbo-like” context of the asylum-seeking situation might be what is hampering the effectiveness of coping resources, due to the situation’s inherent uncertainty and uncontrollability [14]. Given the high levels of post-migratory stress refugees experience and the vulnerabilities that can stem from pre-migratory trauma exposures, this seeming lack of stress-buffering effects of coping warrants further investigation. In fact, our results, combined with the findings from Woltin et al. [13] might be viewed as an indicator of the profound constraints placed on refugees and asylum seekers in certain contexts, and the depleting effect these constraints might have on their resources to cope with stressors in an efficient way. If conflict-proximity plays a moderating role in this relationship, evidence supporting this relationship could have large implications for both the asylum-seeking process, but also how aid/health personnel should organise psychosocial support in refugee camps going forward.

The main aim of the present study was therefore to investigate the potential buffering effect of individual level coping strategies (acceptance and problem-solving) in the relationship between post-migration stressors, mental health and well-being in a population consisting of adult, resettled refugees from Syria, currently residing in Sweden. In terms of conflict proximity, this sample population could be considered similar to the sample in the German refugee camp, described above [13] but experiencing a higher level of controllability due to being resettled in Sweden with official resident permits. With respondents being distal to the conflict in Syria, and possibly experiencing increased levels of controllability after being granted residency in Sweden, we hypothesised that dispositional use of acceptance and problem-solving coping strategies in this sample would constitute a protective factor against post-migration adversities’ mental health consequences.

## Material and methods

### Design and participants

The present study uses cross-sectional data from a questionnaire survey conducted in 2016. Eligible participants consisted of adult refugees from Syria who arrived in Sweden on grounds of asylum, and were resettled between 2011 and 2013. Based on information in the Total Population Registry held by Statistics Sweden, a sampling frame of 9662 individuals was created. From this sampling frame, 4000 individuals were selected using simple random sampling and invited to take part in the study via postal mail. The invitation letter contained information about the study, consent procedures and withdrawal options as well as the actual questionnaire. Of the invited participants, 1215 returned the questionnaire, giving a response rate of about 30%. Descriptive statistics on participants versus nonparticipants can be found in Table 1. The present study is part of a larger research project, “Resiliency, Mental Health and Social Participation”, and we refer to a prior publication for more details on recruitment strategies and procedures, as well as issues concerning potential selection bias and representativity [2].

**Table 1:** Descriptives of participating adult refugees from Syria who resettled in Sweden between 2011 and 2013

	n	Mean	(%, SD or percentile)
Gender, n (%)			
Men	763		(62.8)
Women	452		(37.2)
Age, mean (SD)	1,215	38.7	(11.3)
Education, n (%)			
0–9yrs	453		(38.4)
10–12 yrs	255		(21.6)
13–14 yrs	234		(19.8)
≥ 15y rs	237		(20.1)
Civil status, n (%)			
Married	771		(63.5)
Unmarried	386		(31.8)
Divorced/widower(ed)	58		(4.8)
Year immigration, n (%)			
≤ 2011	76		(6.3)
2012	334		(27.6)
2013	802		(66.2)
PTE-AR fraction*, mean (SD)	1,143	0.38	(0.22)
HSCL-25, mean (SD)	1,181	1.73	(0.59)
WHO-5 index, mean (SD)	1,198	2.88	(1.36)
Post-migration stressors^†^, mean (SD)			
Financial strain	1,192	2.82	(1.26)
Social strain	1,172	2.62	(1.19)
Competency strain	1,181	2.62	(1.09)
Discrimination	1,180	1.63	(0.76)
Coping strategies^‡^, mean (25th/75th percentile)			
Problem-solving	1,168	3.5	(2.5/4.0)
Acceptance	1,196	3.0	(2.5/4.0)

*PTE-AR = Potentially Traumatic Experiences Adversity Ratio fraction = [number of PTEs experienced]/[total number inquired about].

^†^Post-migration stressors = mean-item score for a given domain (e.g., financial strain). All items for all four domains were scored on a 5-point Likert scale going from 1 (Never) to 5 (Very often).

^‡^Coping strategies = mean-item score on four (problem-solving) or two (acceptance) items tapping how frequent coping styles are used, scored on a Likert scale from 1 = “I haven’t been doing this at all” to 4=“I have done this a lot”.

### Measurement

Symptoms of anxiety and depression were measured with the Hopkins Symptoms Checklist [15]. This is one of the most widely used scales to measure anxiety and depression in refugee populations [16], and several studies have supported the scale’s construct validity across populations [17], even if a recent study raises some concern when used in Arabic speaking populations [18]. The scale consists of 25 items—the first 10 tapping into anxiety and the last 15 depression. All 25 items are answered on a 4-point Likert scale ranging from 1 = not at all to 4 = very much. The overall mean-item score on the 25-item scale is frequently used as a combined measure of anxiety and depression, and this approach was utilized in the present study. An overall mean-item score was only calculated for individuals with ≤ 3 missing and was estimated as the HSCL-sum score divided by the number of items answered. Participants with more than three missing items were assigned a missing value on the HSCL mean-item variable.

Subjective well-being (SWB) was measured with the WHO-5 index, which has been extensively used and validated across populations and cultures, including among refugee populations [19, 20]. The 5-item scale is positively framed and each item is scored on the 6-point Likert scale going from 0 = none of the time to 5 = all of the time. A mean-item score was calculated for participants with ≤ 1 missing in the same manner as for HSCL.

Four domains of the Refugee Post-Migration Stress Scale (RPMS) were used to measure stressors related to the post-migratory environment [21]. The four domains were chosen because they tap into stressors related to the host-country environment which is amenable to intervention. The scale has been preliminarily validated among Syrian refugees granted asylum in Sweden [21]. The four domains investigated were: financial strain (e.g. “Worry about unstable financial situation”); social strain (e.g. “ Frustration due to loss of status in the Swedish society”); competency strain (e.g. “ Difficulties understanding how ordinary life activities in Sweden work [shopping, buying tickets, traveling, etc.]”); and perceived discrimination (e.g. “Feeling disrespected due to my national background”). The first three domains consist of three items and the last domain consists of four items. All items are scored on a 5-point Likert scale going from 1 = never to 5 = very often. A mean-item score for each domain was calculated for participants without missing on that domain.

Potentially Traumatic Experiences (PTEs) were measured using the Refugee Trauma History Checklist (RTHC) which consists of 16 items relating to traumatic exposure prior to and during flight [22, 23]. The identical eight PTEs are inquired about before and during flight (e.g., have you experienced war at close quarters/sexual violence/torture). The present study combined the 16 items into one variable operationalized through a PTE-fraction as suggested by Steel et al. [24]. This was calculated as the number of experienced PTEs divided by the total number inquired about (i.e., 16). For example, someone having experienced eight PTEs either pre-flight or during flight were given a PTE-fraction of (0.50).

Coping strategies were measured using the BRIEF Cope scale [25]. The scale consists of 28 questions tapping into 14 coping strategies, each measured with two items. Each item is answered on 4-point Likert scale going from 1 = I haven’t been doing this at all to 4 = I have been doing this a lot. The present study included eight items relating to the coping strategies: planning, active coping, positive reframing and acceptance. In line with theory and prior evidence [26], and supported by supplementary analyses using principal component analysis and factor analysis, the two first strategies (i.e., planning and active coping) were combined into a problem based coping strategy, from hereon referred to as *problem-solving* [26]. In the literature, the positive reframing strategy has in some studies loaded most strongly on a higher-order “problem-based” factor whereas in other studies it has loaded on a higher-order “emotion-focused” factor [27]. Supplementary analyses in the present study also showed significant cross-loading of the positive reframing strategy, thus the two items constituting this strategy were dropped. The two items on acceptance are from hereon referred to as *acceptance* or* acceptance-based coping.*

Education, year for migration and marital status were obtained from self-report data in the questionnaire. Age was modelled continuously; marital status was divided into 3 categories (married/unmarried/divorced or widow(er)); and education was split into four categories: ≤ 9 years/10–12 years/13–14 years/ ≥ 15 years). Year of immigration was categorized into three groups: 2008–2011/2012/2013. The above-described handling of sociodemographic and background variables was done to be consistent with prior publications.

### Statistical analysis

Data was first checked for missing values, errors and outliers using simple cross tabulations and frequency distributions. The number of participants with missing data on individual variables can be inferred from the descriptive table. In regression models, missing were handled through pairwise deletion and the number contributing data to a given model is indicated in relevant tables.

Linear regression analysis with interaction was done to investigate the main aim of the study, namely, whether coping strategies moderated the presumed associations between post-migratory stressors and mental health (symptoms of anxiety/depression and subjective well-being). Regression models were built within the framework of predictive/causal modelling [28], i.e., focusing on the associations between each post-migration stressor and the three mental health outcomes, and whether coping strategies modified these associations. Effect modification was tested using Wald test for interaction, with one interaction tested at a time. That is, a single interaction term between a given coping strategy and post-migration stressor was tested first in fully adjusted models. This was subsequently repeated for all possible combinations of coping strategy, post-migration stressor and mental health outcome (16 total combinations). In order to adjust for multiple testing and the risk of type-I error, p-values from interaction tests were evaluated using the Benjamini–Hochberg procedure [29]. In this procedure, all estimated p-values are ranked in order from smallest to largest and then compared with a critical threshold value estimated based on a pre-determined acceptable risk of making a type-I error and the number of tests undertaken [30]. The acceptable type-I error rate for the present study was set to 5% (with sensitivity analysis done with a 10% error rate). Specifically, starting at the lowest estimated p-value, each p-value is compared to the threshold value and deemed statistically significant if below the threshold. The process is continued until the estimated p-value is higher than the threshold, at which point all subsequent p-values are considered insignificant. Statistically significant interactions were then evaluated further through graphical analysis, with mental health plotted against post-migration stressors across different levels of coping style utilization. Specifically, the 25th (low utilization), 50th (medium utilization) and 75th percentiles (high utilization) was plotted and compared with confidence intervals (CIs). Regression coefficients are presented in both unstandardized (B) and standardized (β) for continuous variables to facilitate comparisons.

## Results

Given that prior publications have presented descriptive statistics on the sample, Table 1, which shows distributional properties of included variables is placed in the Methods section. Compared to the sample frame (N = 4,000), participants (n = 1,215) were older (19.5% vs. 14.6% in highest age-group); had a higher proportion with a university degree (38.7% vs. 31.5%); consisted of more married individuals (63.5% vs. 52.9%); and had a lower proportion who had arrived in 2011 or earlier (6.5% vs. 10.1%). There was, however, no difference in the gender composition [2].

Table 2 shows fully adjusted linear regression models of anxiety/depression symptoms and well-being regressed on post-migration stressors, coping strategies and background variables. Both financial and social strain showed very strong associations with anxiety/depression and wellbeing (p-values < 0.001), with the standardized regression coefficient, β, almost twice as large for financial strain compared to social strain across outcomes. Specifically, for financial strain, a one standard deviation increase in strain was associated with a 0.3 standard deviation increase in anxiety/depression and a 0.3 standard deviation decrease in well-being. Host-country competence strain and discrimination showed more inconsistent associations with mental health. There was no evidence that host-country competency strain was associated with symptoms of anxiety/depression and only weak evidence of an association with well-being. For discrimination on the other hand, there was no evidence of an association with well-being, however, there was strong evidence of an association with symptoms of anxiety/depression (p < 0.001), with β less than half the size of financial strain and somewhat smaller than that of social strain. Both coping strategies investigated were independent predictors of lower anxiety/depression and higher well-being. Gender and PTEs were associated with both outcome variables (with female gender and more PTEs positively linked to anxiety/depression and negatively to well-being), whereas age showed a positive association with anxiety/depression, but none with well-being.

**Table 2:** Fully adjusted linear regression models of HSCL and WHO well-being index without interaction

	HSCL mean score				Well-being mean score			
	B	(95% CI)	β	*p*	B	(95% CI)	β	*p*
Post-migration stressor								
Financial strain	0.13	(0.11 to 0.16)	0.29	***	−0.32	(−0.39 to −0.25)	−0.30	***
Social strain	0.09	(0.06 to 0.12)	0.18	***	−0.18	(−0.25 to −0.10)	−0.16	***
Host country comp. strain	0.02	(−0.01 to 0.05)	0.04		−0.09	(−0.16 to −0.02)	−0.07	*
Discrimination	0.10	(0.06 to 0.14)	0.13	***	−0.09	(−0.19 to 0.01)	−0.05	
Coping strategy								
Problem-based coping	−0.09	(−0.13 to −0.04)	−0.10	***	0.18	(0.08 to 0.29)	0.09	**
Emotion-based coping	−0.10	(−0.13 to −0.06)	−0.14	***	0.31	(0.22 to 0.39)	0.19	***
Gender (female vs. male)	0.16	(0.11 to 0.22)	NA	***	−0.21	(−0.36 to −0.06)	NA	**
Age	0.01	(0.00 to 0.01)	0.13	***	−0.01	(−0.01 to 0.00)	−0.05	
Marital status (vs. married)								
Unmarried	0.16	(0.09 to 0.23)	NA	***	−0.32	(−0.50 to −0.14)	NA	***
Divorced/widow(er)	0.23	(0.10 to 0.36)	NA	**	−0.17	(−0.50 to 0.15)	NA	
Education (vs. ≤ 9 years)								
10–12 years	0.03	(−0.04 to 0.10)	NA		−0.14	(−0.32 to 0.04)	NA	
13–14 years	−0.01	(−0.08 to 0.07)	NA		−0.19	(−0.38 to 0.00)	NA	
≥ 15 years	−0.00	(−0.08 to 0.07)	NA		−0.33	(−0.52 to −0.14)	NA	**
Year immigration (≤ 2011)								
2012	−0.09	(−0.21 to 0.03)	NA		0.35	(0.05 to 0.66)	NA	*
2013	−0.18	(−0.29 to −0.07)	NA	**	0.41	(0.12 to 0.70)	NA	**
PTE-AR fraction	0.48	(0.35 to 0.61)	0.19	***	−0.54	(−0.87 to −0.22)	−0.09	**

All variables in first column were included in linear regression model. B = unstandardized regression coefficient. β = standardized regression coefficient

***p < 0.001; **p < 0.01; *p < 0.05

Table 3 shows all tested interactions between coping and post-migration stressors (16 interactions in total given by all possible combinations of coping style, post-migration stressor and mental health outcome). Interactions are presented in ranked ascending order based on p-values (i.e., smallest on top and largest at bottom). After adjusting for multiple testing through the Benjamini Hochberg procedure with a 5% false positive threshold, there were three statistically significant interactions, all with anxiety/depression as the outcome (in fact, using a 10% false positive threshold did not result in more interactions being significant). Two of the interactions involved financial strain, meaning both problem-solving and acceptance strategies moderated the associations between financial strain and anxiety/depression. The last statistically significant interaction was that problem-solving strategies moderated the association between host-country competence strain and anxiety/depression. When the interaction analyses were repeated with the sample split by gender, the same overall narrative emerged for males and females, that is, the three interactions with the lowest p-values were identical across gender. None of the coping strategies moderated any of the adverse associations between post-migration stressors and well-being.

**Table 3:** All tested interactions between post-migration stressors and coping styles, with Benjamini–Hochberg critical values

Interaction tested	Outcome variable	Rank	p-value*	B-H critical value (0.05)^†^	B-H critical value (0.10)^‡^
problem-solving * financial strain	***HSCL mean score***	***1***	***0.000***	***0.003***	***0.006***
acceptance * financial strain	***HSCL mean score***	***2***	***0.004***	***0.006***	***0.013***
problem-solving * host country competency strain	***HSCL mean score***	***3***	***0.004***	***0.009***	***0.019***
problem-solving * discrimination	Well-being mean score	4	0.044	0.013	0.025
acceptance * discrimination	Well-being mean score	5	0.090	0.016	0.031
acceptance * social strain	Well-being mean score	6	0.175	0.019	0.038
acceptance * host country competency strain	Well-being mean score	7	0.190	0.022	0.044
problem-solving * social strain	HSCL mean score	8	0.200	0.025	0.050
acceptance * discrimination	HSCL mean score	9	0.250	0.028	0.056
acceptance * social strain	HSCL mean score	10	0.289	0.031	0.063
problem-solving * host country competency strain	Well-being mean score	11	0.364	0.034	0.069
problem-solving * discrimination	HSCL mean score	12	0.411	0.038	0.075
acceptance * host country competency strain	HSCL mean score	13	0.472	0.041	0.081
problem-solving * social strain	Well-being mean score	14	0.742	0.044	0.088
acceptance * financial strain	Well-being mean score	15	0.769	0.047	0.094
problem-solving * financial strain	Well-being mean score	16	0.785	0.050	0.100

*p-value from Wald test of interactions in fully adjusted models. Interaction terms were tested sequentially (i.e. one at a time)

^†‡^Benjamini–Hochberg critical value if false positive threshold is set at 5% and 10%, respectively. Boldface + italics indicate that critical value is above actual p-value (i.e., interaction is considered statistically significant after adjustment for multiple testing)

**Fig. 1:**
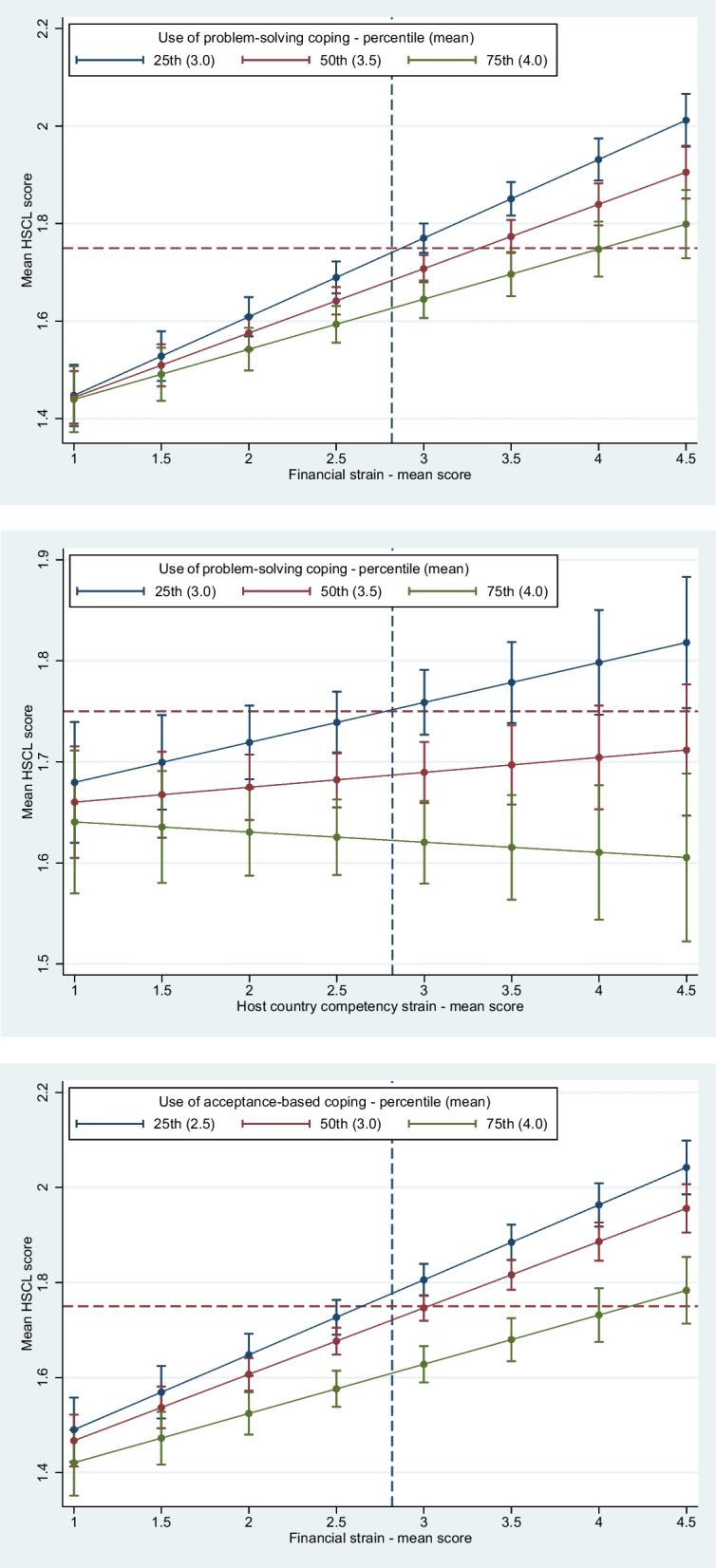
Graphical presentation of statistically significant interactions between coping style and post-migration stressors

Figure 1 gives a graphical presentation of the three statistically significant interactions between coping styles and post-migration stressors with anxiety/depression as the outcome. Graphically, interaction is indicated by the differences in slope between levels of coping style utilization, with the three coloured lines in each graph representing low (blue), medium (red) and high (green) utilization, respectively. As can be seen in all the graphs in the figure, at the mean level of experienced post-migration stress in the sample (vertical blue dotted line), there was a statistically significant difference in the symptom level of anxiety/depression between the 75th and 25th percentile of coping strategy utilization (no overlap in CIs). That is, respondents who scored in the 75th percentile for a given coping strategy (high utilizers of that coping strategy—i.e., green line) had a significantly lower HSCL-score compared to those who scored in the 25th (low utilizers of that coping strategy—i.e., blue line). At lower levels of strain, the confidence intervals were overlapping, suggesting weak/no evidence of a difference in anxiety/depression symptoms comparing high vs. low utilizers, whereas at higher levels of strain, CIs were not overlapping suggesting a statistically significant difference.

## Discussion

The main aim of the present study was to investigate whether problem-solving and acceptance coping strategies moderated (i.e., buffered) the known adverse relationship between post-migration stressors, mental health and well-being, in a population of adult refugees from Syria currently residing in Sweden. A priori, we hypothesised that respondents who dispositionally used problem-solving and acceptance strategies in order to cope with the adversities of resettlement would report higher levels of mental health and well-being, due to the buffering effect of these coping strategies on the relationship between post-migration stress and mental health and well-being. Moreover, when being distal to the conflict in Syria and experiencing increased levels of controllability after being granted residency in Sweden, the potential for success when utilising problem-solving and acceptance strategies should be more pronounced. In other words, utilising coping strategies in a more controlled context might alleviate the known negative association between post-migration stressors and mental health and well-being *to a larger degree* than in more proximal contexts, where controllability and the potential for success might be low, as alluded in our previous study focusing on asylum-seekers [14].

Problem-solving strategies refers here to strategies aimed at directly addressing the source of stress or problem. These types of coping strategies have been linked to improved mental health outcomes, as they empower individuals to take active steps to resolve their problems and improve their situation. By actively addressing the root causes of stressors, problem-solving strategies can help individuals reduce the negative impact that stress has on their mental health. Similarly, acceptance strategies refer here to cognitive strategies aimed at managing and regulating responses to stress by accepting and learning to live with the realities of the situation as they are [26]. Acceptance can therefore play an important role in promoting overall well-being and mental health in adverse situations.

In line with this, our results suggest that both problem-solving and acceptance coping strategies did indeed buffer the adverse association between financial strain and symptoms of anxiety and depression. Furthermore, problem-solving strategies seemed to moderate the association between host-country competency strain and anxiety and depression. In fact, the results indicate that there was no association between host-country competency strain and anxiety/depression among high utilizers of problem-solving coping (top 25th percentile), whereas there was a clear positive and statistically significant association among low utilizers (bottom 25th percentile). This lack of associations among the high utilizers suggests that problem-solving may indeed constitute an effective buffer for the distressing emotional reactions to the lack of important skills needed in the host country. Problem-solving, consisting of planning and active coping, thus, may provide a positive outlook and optimistic expectancies of being able to acquire host country competencies in near future.

Furthermore, acceptance coping showed a similar moderating effect on the associations between financial strain and depression/anxiety. Low utilizers of this coping strategy displayed stronger associations than did the high utilizers. Cognitive reframing strategies such as acceptance have been previously suggested to alleviate emotional distress linked to unemployment by providing a short-term stress relieving mechanism, as well as a long-term adaptation [31]. Thus, for refugees, acceptance may attenuate the distressing emotional short-term response in a life-situation that might be subjectively perceived as transient. Our results, however, do not provide information about the role of acceptance in a sustained financially strained living situation.

Despite the displayed moderating role of coping in regard to anxiety/depression, neither problem-solving or acceptance strategies moderated the negative relationship between post-migration stressors and well-being. Psychological wellbeing and distress could be viewed as both distinct dimensions and/or opposites of a single continuum [32, 33]. Although we found associations of both post-migratory stressors and coping strategies with wellbeing, the coping strategies did not display a buffering function for wellbeing against the included stressors. The results can be seen as suggesting that whereas coping strategies may reduce refugees’ distress-response to post-migratory adversities, wellbeing is not necessarily boosted by this buffer of distress.

Taken together, our findings show that coping strategies did buffer the impact of some of the post migratory stressors included in this study. Although this pattern was not replicated in regard to subjective well-being, these findings show that individual-level coping skills among refugees may contribute to adaptation in the face of post-settlement difficulties and, thus, could be suitable targets for interventions. In line with our findings, refugees should benefit from interventions that include elements from acceptances and commitment therapy, e.g., WHO’s Self‐Help Plus [34]. Interventions which incorporate these elements have been found to be effective in preventing mental ill-health and could be scaled up in refugee populations exposed to ongoing adversities during forced migration or in the resettlement phase in parallel with services that address refugees social and financial needs.

### Limitations

Although the present study includes random sampling from population-based registries, a large sample size and the use of well-established measures of key variables, several limitations need to be mentioned. The cross-sectional design of the study places clear limits on causal interpretations. This point is particularly relevant in light of the recent findings by Wu et. al. [35] which suggest that the effects of post-migration stress on mental health are time-varying. Moreover, the self-reported mental health measures utilized do not investigate other comorbid conditions that may produce similar symptoms or capture the complexity of all mental health dimensions. Nonetheless, these measures have been extensively used and validated to capture essential aspects of coping, mental health and well-being, while keeping questions at a minimum. Relatedly, the PTE-AR measure used should be considered a coarse measure of exposure to PTEs, as it is not obvious that each PTE should have the same weight when investigating PTEs as risk factors for mental health outcomes (e.g., personally experiencing sexual violence is likely a more traumatizing experience than experiencing “war at close quarters”). Finally, there was a fairly high proportion who maxed out on the coping measures used—around 30% for both problem-based and acceptance coping strategies, and this may have resulted in a ceiling effect which could have influenced finding (e.g. attenuated interaction findings).

It is also important to note that the study focused exclusively on refugees from Syria resettled in Sweden between 2011 and 2013. Considering this, the findings may not be generalized to other refugee populations, or even to Syrian refugees coming at different time-periods as the profile of refugee cohorts from Syria may vary between time-periods.

Lastly, no pre-registered study protocol with a detailed analysis plan exists, and analytic choices were partly made with data at hand. For example, the present study did not include any a priori hypothesis as to whether the modifying role of coping strategies would be constant across gender, thus the preliminary finding suggesting gender invariance, therefore, needs to be investigated more thoroughly in future studies. Other parts of the study were broadly conceptualized prior to approaching the data and based on previous evidence and relevant theory.

## Conclusions

The main aim of the present study was to investigate whether problem-solving and acceptance coping strategies moderated the known adverse relationship between post-migration stressors, mental health and well-being, in a population of adult refugees from Syria currently residing in Sweden. In conclusion, our findings show that coping strategies to some degree buffer the impact of financial strain and host-country competency strain experienced in the resettlement phase. Although this pattern was not found in regard to subjective well-being, these findings show that individual-level coping skills among refugees may contribute to adaptation in the face of post-settlement adversities. Notwithstanding the importance of attending to refugees’ psychosocial conditions, refugees residing in refugee camps and newly resettled refugees might benefit from interventions aiming at enhancing individual coping resources and skills. Which factors, except from geographical distance, that constitute markers of conflict proximity with potential effects on controllability and uncertainty also warrant further exploration in future studies.

## Data Availability

The statistical code (Stata) for the analysis in the manuscript is available from the corresponding author. Under Swedish law and ethical approval, individual level data of this kind cannot be publicly available. Individual level data can be made available on reasonable request as long as it is in line with Swedish law and ethical approvals. Aggregated data are available from the corresponding author (upon reasonable request).

## Abbreviations

CI: Confidence interval
HSCL: Hopkins Symptoms Checklist
PTSD: Posttraumatic stress disorder
PTE: Potentially traumatic experiences
SWB: Subjective well-being
